# Genetic variants associated with lung function: the long life family study

**DOI:** 10.1186/s12931-014-0134-x

**Published:** 2014-11-01

**Authors:** Bharat Thyagarajan, Mary Wojczynski, Ryan L Minster, Jason Sanders, Sandra Barral, Lene Christiansen, R Graham Barr, CHARGE consortium, SpiroMeta consortium, Anne Newman

**Affiliations:** Department of Laboratory Medicine and Pathology, University of Minnesota, 515 Delaware Street SE, 1-136 Moos Towers, Minneapolis, MN 55455 USA; Department of Genetics, Division of Statistical Genomics, Washington University, 4444 Forest Park Parkway, CB#8506, St. Louis, MO USA; Department of Human Genetics, University of Pittsburgh, 728 Parran Hall, Pittsburgh, PA 15261 USA; Department of Epidemiology, Center for Aging and Population Health, University of Pittsburgh, 130 De Soto Street, Pittsburgh, PA 15261 USA; Department of Neurology, Columbia University, G.H. Sergievsky Center, 630 W 168th St, P&S 16, New York, NY 10032 USA; Department of Epidemiology, Biostatistics and Biodemography, University of Southern Denmark, J. B. Winsløws Vej 9 B, st. th, 5000 Odense, C Denmark; Department of Medicine, Columbia University Medical Center, PH 9 East, Room 105, New York, NY 10032 USA; Department of Epidemiology, Columbia University Medical Center, PH 9 East, Room 105, New York, NY 10032 USA

**Keywords:** GWAS, Lung function, Aging

## Abstract

**Background:**

Reduced forced expiratory volume in 1 second (FEV_1_) and the ratio of FEV_1_ to forced vital capacity (FVC) are strong predictors of mortality and lung function is higher among individuals with exceptional longevity. However, genetic factors associated with lung function in individuals with exceptional longevity have not been identified.

**Method:**

We conducted a genome wide association study (GWAS) to identify novel genetic variants associated with lung function in the Long Life Family Study (LLFS) (n = 3,899). Replication was performed using data from the CHARGE/SpiroMeta consortia. The association between SNPs and FEV_1_ and FEV_1_/FVC was analyzed using a linear mixed effects model adjusted for age, age^2^, sex, height, field center, ancestry principal components and kinship structure to adjust for family relationships separately for ever smokers and never smokers. In the linkage analysis, we used the residuals of the FEV_1_ and FEV_1_/FVC, adjusted for age, sex, height, ancestry principal components (PCs), smoking status, pack-years, and field center.

**Results:**

We identified nine SNPs in strong linkage disequilibrium in the *CYP2U1* gene to be associated with FEV_1_ and a novel SNP (rs889574) associated with FEV_1_/FVC, none of which were replicated in the CHARGE/SpiroMeta consortia. Using linkage analysis, we identified a novel linkage peak in chromosome 2 at 219 cM for FEV_1_/FVC (LOD: 3.29) and confirmed a previously reported linkage peak in chromosome 6 at 28 cM (LOD: 3.33) for FEV_1_.

**Conclusion:**

Future studies need to identify the rare genetic variants underlying the linkage peak in chromosome 6 for FEV_1_.

**Electronic supplementary material:**

The online version of this article (doi:10.1186/s12931-014-0134-x) contains supplementary material, which is available to authorized users.

## Introduction

Pulmonary function, as determined by spirometry, indicates the physiological state of the airways and lung. Pulmonary function measures such as forced expiratory volume in one second (FEV_1_), forced vital capacity (FVC) and the ratio of the two measures (FEV_1_/FVC) are used to diagnose chronic obstructive pulmonary disease (COPD) and assist in the diagnosis of asthma, which are major causes of death and morbidity worldwide [1]. Reduced (FEV_1_) and forced vital capacity (FVC), in healthy asymptomatic adults, are predictors of cardiovascular disease mortality and all cause mortality, independent of smoking history [2-9]. The Danish 1905 cohort study also showed that higher FEV_1_ and peak expiratory flow (PEF) in nonagenarians to be associated with lower mortality [10]. Due to the strong and consistent association with mortality, pulmonary function has been viewed as a biomarker of aging itself [11]. We have previously shown that the prevalence of self-reported COPD was 3 times lower in the Long Life Family Study (LLFS) as compared to the other similarly aged cohorts [12] suggesting that LLFS participants, who were selected for exceptional familial longevity, may have better lung function as compared to the general population. These findings are supported by a previous study that reported male offspring of long lived parents had higher lung function as compared to those with short lived parents [13]. Several studies have shown that pulmonary function measures are heritable characteristics with estimates ranging from 38% for FEV_1_ to 37% for FEV_1_/FVC [14-16]. However, the 26 genetic loci associated with FEV_1_/FVC, FEV_1_ or both (23 loci associated with FEV_1_/FVC and 10 loci associated with FEV_1_) [17-19] in genome wide association studies explain only around 3% of the variance in FEV_1_/FVC and around 1.5% of the variance in FEV_1_ [18]. Furthermore, the mean age of Cohorts for Heart and Aging Research in Genomic Epidemiology (CHARGE)/SpiroMeta consortia was 55 years; hence, it identified genes for lung function among older adults. Since findings from the LLFS [12] and a previous study [13] suggest that there may be genetic determinants of lung function among exceptionally long lived individuals and families, we conducted a genome wide association and linkage study among participants of the LLFS, a family based cohort of exceptional longevity, to identify novel genetic determinants of lung function in this unique sample.

## Material and methods

### Cohort description and study design

The LLFS study design has also been described in detail previously [12]. Briefly, the LLFS is a family-based cohort study (n = 4,559) that enrolled long-lived probands and their siblings (n = 1,445), their offspring (n = 2,329) and spousal controls (n = 785) recruited from 3 U.S. field centers (Boston University Medical Center in Boston MA, Columbia College of Physicians and Surgeons in New York City NY, and the University of Pittsburgh in Pittsburgh PA) and the University of Southern Denmark to identify genetic determinants of longevity in these families. At the U.S. field centers, an initial recruitment brochure was mailed to all people in the Center for Medicare and Medicaid Services list of Medicare enrollees who were ≥89 years old on January 1, 2005, were not in end stage renal disease or hospice programs and lived in zip codes within 3 hours driving distance of one of the three U.S field centers. Mailings were conducted in collaboration with CMS and the NIA via an Intra-Agency Agreement. Study participants were also recruited from the local communities using mailed brochures, posters, web-based media and newspaper advertisements as well as community presentations at churches and senior centers. Additional mailing lists were obtained through local government agencies or purchased public domain lists from commercial vendors. The University of Southern Denmark used the Danish National Register of Persons to identify individuals who were ≥90 years during the study recruitment period without any restrictions on residence [20]. Only families who had the proband, at least one living sibling, and one of their living offspring (minimum family size of 3) with a Family Longevity Selection Score (FLoSS) of 7 or higher that correlates well with later-observed longevity [21] and gave informed consent and were willing to participate in the interview and examination including donating a blood sample were eligible to participate in this study. This strategy led to the enrollment of families with the greatest potential utility for phenotypic and genetic studies of exceptional survival in families. All research was performed in compliance with the Helsinki Declaration. Written informed consent was obtained from all enrollees. In a few cases of cognitive impairment, family members were enrolled via proxy consent, provided that the participant was able to express assent at the time of the examination. This study was approved by the Institutional Review Boards at all the institutions in the United States of America and Denmark.

After excluding 15% of the participants due to presence of non-European ancestry (n = 6), low quality spirometry (defined as 2 or more acceptable spirometry maneuvers with reproducibility within 250 mL) (n = 295), self-reported pulmonary fibrosis (n = 11) obtained during an in-person interview, history of lung volume reduction surgery (n = 14), or missing genotypes (n = 344), a total of 3,889 participants were included in the present analysis.

### Lung function measurements

The examinations were conducted in the home setting with portable equipment by centrally trained and certified research assistants using a standardized protocol. Lung function was measured with a portable spirometer (EasyOne Diagnostic, NDD Medical Technologies, Andover, MA) following American Thoracic Society guidelines [22]. Calibration checks and the best 3 maneuvers were reviewed centrally by one investigator. Only spirometry tests with 2 or more acceptable maneuvers with reproducibility within 250 mL were selected for further data analyses.

### Genotyping and imputation

The Human Omni chip 2.5 v1 (Illumina Inc., Ca), was used to genotype all the LLFS participants at the Center for Inherited Disease Research (CIDR). Ancestry principal components (PCs), to control for population structure, were produced with EIGENSTRAT [23] on 1,515 LLFS unrelated individuals using 120,093 tag SNPs, where in advance any SNPs with minor allele frequencies (MAF) <5%, Hardy Weinberg Equilibrium (HWE) *p* <1E-06, and with missing genotypes were excluded. Ancestry PCs produced from unrelated subjects were expanded, within EIGENSTRAT framework, to all members of LLFS. Genotype imputations were performed based on the cosmopolitan phased haplotypes of 1000 Human Genome (1000HG, version 2010–11 data freeze, 2012-03-04 haplotypes) using MACH and MINIMACH [24,25] and a total of 38,045,518 SNPs were imputed. When MAF ≥ 0.05 and r^2^ > 0.3 for imputed SNP filters were applied to the hybrid dataset for analysis, the number of SNPs for analysis is reduced to 6,522,421 (from a total of 38,245,546 SNPs), of which 1,204,935 SNPs were genotyped and 5,317,486 SNPs were imputed.

### Statistical analysis

The statistical models used to test the association between the GWAS SNPs and lung function (FEV_1_ and FEV_1_/FVC) were identical to the models used by the CHARGE/SpiroMeta consortia [18], except that the LLFS study also included adjustment for kinship structure to facilitate replication of results in the CHARGE/SpiroMeta consortia. We employed a linear mixed effects model which adjusted for age, age^2^, sex, height, field center and ancestry PCs (PC1-20) in addition to the kinship matrix. The adjusted phenotypic residuals from these models (FEV_1_ and FEV_1_/FVC) were inverse normal transformed to normally distributed *z*-scores. These transformed residuals were then used as the phenotype for association testing under an additive genetic model, separately for ever smokers and never smokers. The associations between individual SNPs the FEV_1_ (milliliters) and FEV_1_/FVC (percent) were analyzed using a linear mixed effects model with kinship structure [26,27] to adjust for family relationships separately for ever smokers and never smokers. The effect estimates and standard errors for ever-smokers and never smokers were meta-analyzed using inverse-variance weighting.

We used the same criteria as the CHARGE/SpiroMeta consortia for assessing genetic associations [18]. All SNPs that showed a borderline association (p < 5E-06) with FEV_1_ and FEV_1_/FVC in LLFS were evaluated for their association with FEV_1_ and FEV_1_/FVC in the CHARGE/SpiroMeta consortia [18] using statistical models identical to those described above (individual family-based studies within the CHARGE/SpiroMeta did adjust for kinship structure while other studies within the consortia did not adjust for kinship structure). Finally, genotypes from both LLFS and CHARGE/SpiroMeta were meta-analyzed using METAL to evaluate the overall association between individual SNPs and lung function in both studies. We also evaluated the replication of previously reported GWAS hits (from the CHARGE/SpiroMeta consortia) for FEV_1_ and FEV_1_/FVC in the LLFS population using the statistical models described above.

To calculate Identity by Descent (IBD) for the linkage analyses, the ZAPLO program was used to estimate haplotypes of SNPs in small regions (0.5 cM intervals) [28]. The deCODE map was used to approximate the cM positions [29]. We identified all SNPs in our GWAS scan with no Mendel inconsistencies and an average pedigree heterozygosity ≥0.1. Within each 0.5 cM interval we used the first five such SNPs to construct a haplotype and if there were fewer than 5, we took all such SNPs in the interval. With the resulting haplotypes, IBD at 1 cM intervals was estimated in the Loki program [30], which does chromosome-wide IBD estimation in intact pedigrees. These IBD estimates were then used in the SOLAR package [31] to conduct a variance-component linkage analysis. In the linkage analysis, we used the residuals of the FEV_1_ and FEV_1_/FVC phenotypes, adjusted for age, sex, height, ancestry PCs, smoking status (current, former, never), pack-years, and field center. The SOLAR package was also used to estimate heritability and empirical *p* of LOD.

## Results

There were 1,734 (45%) male participants and 2155 (55%) female participants with an average age of 68.6 years (standard deviation: 15.2 years) and an average BMI of 27.13 kg/m^2^ (standard deviation: 4.79 Kg/m^2^). There were 2,203 (57%) never smokers, 1,403 (40%) former smokers and 283 (3%) current smokers. The average number of cigarettes smoked among former smokers was 20.26 pack years (standard deviation: 22.07 pack years) while the average number of cigarettes smoked among current smokers was 28.25 pack years (standard deviation:19.03 pack years). There were 89 participants (2.3%) with self-reported history of chronic obstructive pulmonary disease (COPD) and 339 (8.7%) participants with self-reported history of asthma, 123 (3.1%) participants with a self-reported history of congestive heart failure and 11 (0.28%) participants with a self reported history of lung cancer. As shown in Table 1, the LLFS population was significantly older (68.6 ± 15.2 years vs. 53.5 ± 7.7 years; p < 0.0001) and had significantly higher percent never-smokers (57% vs. 42%; p < 0.0001) as compared to CHARGE/SpiroMeta consortia. In addition, LLFS had slightly lower FEV_1_ (2455 ml vs. 2963; p < 0.0001) and FEV_1_/FVC (0.76 vs. 0.78; p < 0.0001) as compared to the CHARGE/SpiroMeta consortia (Table 1).

**Table 1:** **Comparison of demographic and lung function variables** ^*^ **in LLFS and CHARGE/SpiroMeta consortia** ^**+**^

	**LLFS (n = 3889)**	**CHARGE/SpiroMeta (n = 48201)**	**p value**
Age (years)	68.6 (15.2)	53.5 (7.7)	<0.0001
Sex (% Male)	44	44	0.62
Smoking Status (% never smoker)	56.6	42.0	<0.0001
FEV_1_ (ml)	2455 (866)	2963 (798)	<0.0001
FEV_1_/FVC	0.76 (0.07)	0.78 (0.09)	<0.0001

^*^Values are means (standard deviations in parentheses) or percentages.

^+^Values for the demographic and lung function variables in the CHARGE/SpiroMeta consortia were calculated using previously published data (Additional file 2: Table S1 a in Soler Artigas et al.) [18].

We evaluated 6,522,421 SNPs across 3,889 individuals. The Q-Q plots for FEV_1_ and FEV_1_/FVC are shown in Additional file 1: Figures S1a and S1b respectively, while the Manhanttan plots for FEV_1_ and FEV_1_/FVC are shown in Additional file 1: Figures S2a and S2b respectively. Overall, we found 130 SNPs (23 genotyped SNPs and 107 imputed SNPs) that showed borderline association with FEV_1_ (p < 5E-06) (Additional file 2: Table S1) and 74 SNPs (14 genotyped SNPs and 60 imputed SNPs) that showed borderline association with FEV_1_/FVC (p < 5E-06) (Additional file 2: Table S2). There was one SNP, rs71374110 in the *ANKRD11* gene that showed a borderline association with both FEV_1_ and FEV_1_/FVC (Additional file 2: Tables S1 and S2). Among the GWAS SNPs with borderline association, 49 SNPs for FEV_1_ and 28 SNPs for FEV_1_/FVC were available for replication in the CHARGE/SpiroMeta dataset (Additional file 2: Tables S3 and S4 respectively). The results for the GWAS SNPs with p <9.0E-07 are presented in Tables 2 and 3 and the data for all SNPs are presented in Additional file 2: Tables S1 and S2. As shown in Table 2, rs1493131 in the *CYP2U1* gene showed borderline association with FEV_1_ (p = 7.4E-07). In addition, 7 imputed SNPs in the *CYP2U1* gene and 1 imputed SNP in the *PHACTR2* gene also showed a borderline association with FEV_1_ (p < 9.2E-07). However, none of these SNPs were associated with FEV_1_ in the CHARGE/SpiroMeta consortia or in the overall meta-analysis. Five of the 9 previously identified GWAS SNPs (p < 1E-07) for FEV_1_ were nominally associated with FEV_1_ in LLFS (p < 0.05) (Additional file 2: Table S5). For FEV_1_/FVC, one SNP, rs889574 in the *ANKRD11* gene, demonstrated a borderline association (p = 1.6E-07) in the LLFS GWAS, (Table 3). Seven of 22 previously identified GWAS SNPs (p < 1E-07) were also associated with FEV_1_/FVC in the LLFS (p < 0.05) (Additional file 2: Table S6). A complete list of all SNPs associated with lung function (p < 5E-06) is shown in Additional file 2: Tables S1 and S2, with their annotation and quality control information.

**Table 2:** **GWAS findings for FEV**_**1**_ **in the LLFS (p <1E-07) and replication in the CHARGE/SpiroMeta consortia**

**LLFS**							**CHARGE/SPIROMETA CONSORTIA**			**META ANALYSIS**				
**SNP**	**Chromosomal position**	**Gene**	**Coded/non-coded allele**	**Coded allele frequency**	**β (SE)**	**P_value**	**Coded allele frequency**	**β (SE)**	**P_value**	**Coded allele frequency**	**β (SE)**	**P_value**	**HetPVal**	**N**
rs9390140	Chr 6: 144127026	*PHACTR2*	C/G	0.33	−0.121(0.025)	8.83E-07	0.33	0.002(0.008)	0.78	0.33	−0.008(0.007)	0.25	1.80E-06	51073
rs1493126	Chr 4:108855828	*CYP2U1*	C/G	0.23	0.139(0.028)	8.24E-07	0.22	−0.005(0.009)	0.53	0.22	0.007(0.008)	0.42	9.48E-07	51687
rs4956031*	Chr4: 108857140	*CYP2U1*	T/C	0.23	0.138(0.028)	9.17E-07	0.22	−0.005(0.009)	0.52	0.22	0.007(0.008)	0.43	1.04E-06	51715
rs998405	Chr4: 108862837	*CYP2U1*	C/G	0.23	0.139(0.028)	7.93E-07	0.22	−0.006(0.009)	0.50	0.22	0.006(0.008)	0.43	8.18E-07	51501
rs17564501	Chr4: 108863209	*CYP2U1*	A/C	0.77	−0.139(0.028)	7.93E-07	0.78	0.006(0.009)	0.50	0.78	−0.006(0.008)	0.44	8.15E-07	51456
rs1493122	Chr4: 108864651	*CYP2U1*	T/C	0.77	−0.139(0.028)	7.59E-07	0.79	0.006(0.009)	0.47	0.78	−0.006(0.008)	0.46	7.40E-07	51105
rs1493131*	Chr4: 108860906	*CYP2U1*	A/G	0.23	0.139(0.028)	7.37E-07	0.22	−0.006(0.009)	0.47	0.22	0.006(0.008)	0.46	7.23E-07	51877
rs11724895	Chr4: 108865791	*CYP2U1*	C/G	0.77	−0.139(0.028)	7.72E-07	0.79	0.006(0.009)	0.46	0.78	−0.006(0.008)	0.47	8.06E-07	51068
rs17564543	Chr4: 108863481	*CYP2U1*	T/C	0.77	−0.138(0.028)	9.07E-07	0.78	0.007(0.009)	0.40	0.78	−0.005(0.008)	0.53	7.47E-07	50935

*These SNPs were genotyped using the Human Omni chip 2.5 v1 (Illumina Inc., Ca). Other SNPs were imputed.

**Table 3:** **GWAS findings for FEV**_**1**_ **/FVC in the LLFS (p <1E-07) and replication in the CHARGE/SpiroMeta consortia**

**LLFS**							**CHARGE/SPIROMETA CONSORTIA**			**META ANALYSIS**				
**SNP**	**Chromosomal position**	**Gene**	**Coded/non-coded allele**	**Coded allele frequency**	**β (SE)**	**P_value**	**Coded allele frequency**	**β (SE)**	**P_value**	**Coded allele frequency**	**β (SE)**	**P_value**	**HetPVal**	**N**
rs889574*	Chr16: 89386808	*ANKRD11*	T/C	0.31	0.129(0.025)	1.63E-07	0.34	0.003(0.007)	0.67	0.66	−0.013(0.007)	0.06	9.99E-07	50425

*These SNPs were genotyped using the Human Omni chip 2.5 v1 (Illumina Inc., Ca). Other SNPs were imputed.

**Figure 1:**
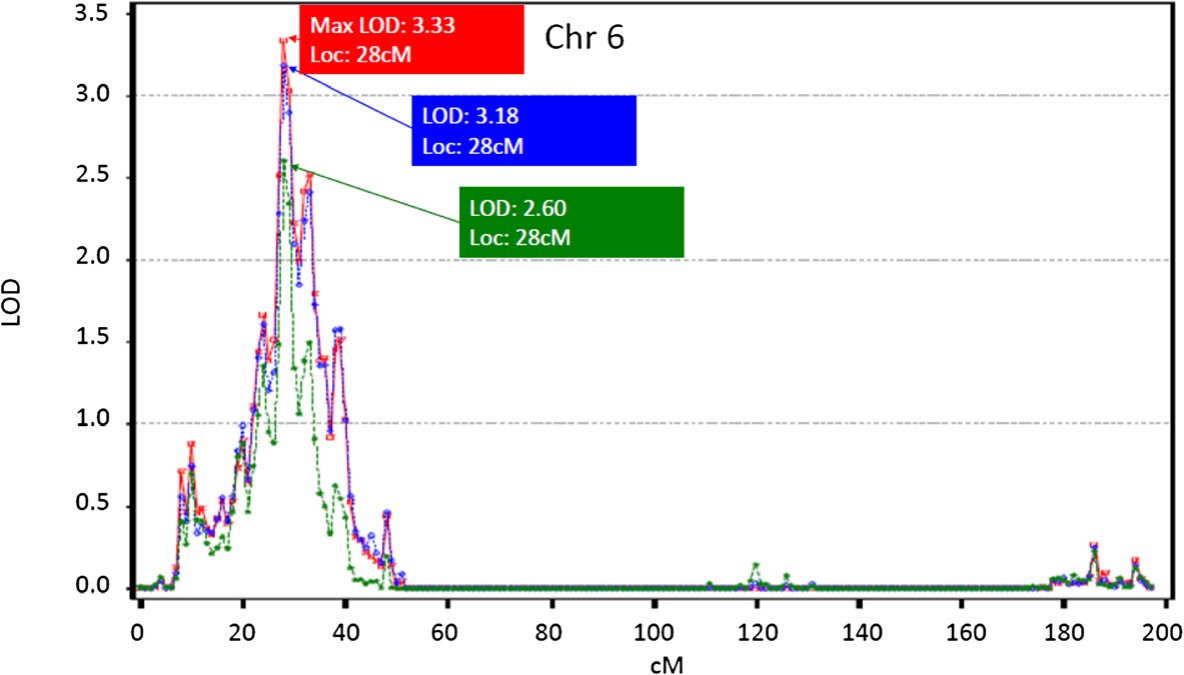
**Linkage peaks on chromosome 6 associated with FEV**_**1**_ **before and after adjustment for GWAS SNPs under the linkage peak associated with FEV**_**1**_ **.** Original linkage is in red, the linkage in blue is for adjustment of GWAS SNPs (n = 3) in a narrow region from 26–34 cM that were associated with p < 1E-03, and green is the linkage after adjustment of GWAS SNPs (n = 19) in a broad region from 10–50 cM that were associated with p < 1E-03.

**Figure 2:**
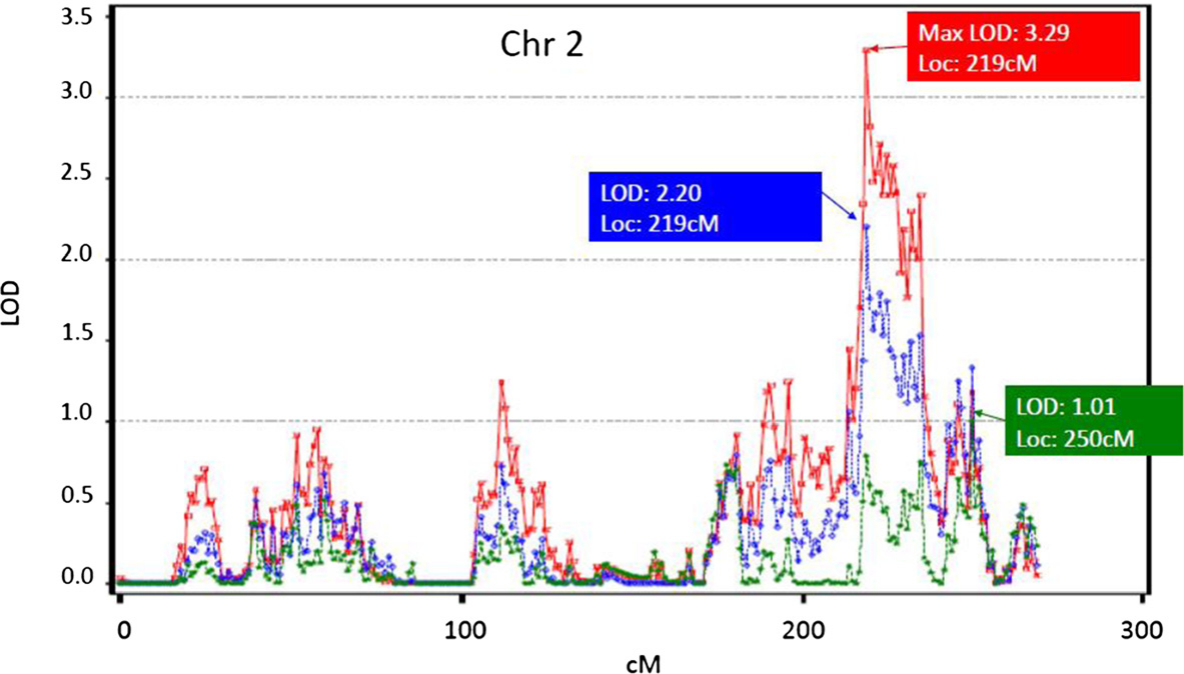
**Linkage peaks on chromosome 2 associated with FEV**_**1**_ **/FVC before and after adjustment for GWAS SNPs under the linkage peak associated with FEV**_**1**_ **/FVC.** Original linkage is in red, the linkage in blue is for adjustment of GWAS SNPs (n = 10) in a narrow region from 217–236 cM that were associated with p < 1E-03, and green is the linkage after adjustment of GWAS SNPs (n = 38) in a broad region from 200–250 cM that were associated with p < 1E-03.

The heritability of FEV_1_ and FEV_1_/FVC as estimated by the SOLAR package was 0.37 ± 0.043 and 0.34 ± 0.040 respectively in the LLFS. The highest LOD score obtained for FEV_1_ was 3.33 localized to 28 cM (chr 6: 9275152 bp – 9467267 bp) at the q terminus of chromosome 6 (Figure 1). This locus remained significant (LOD = 3.18) even after adjustment for 3 GWAS SNPs/indels located between 26 cM-34 cM on chromosome 6 and were nominally associated with FEV_1_ (p < 0.001) (Additional file 2: Table S7). A more comprehensive adjustment for 19 GWAS SNPs that were nominally associated with FEV_1_ (p < 1E-03) over a larger range (10 cM – 50 cM) further attenuated the linkage peak (LOD = 2.60) but did not completely explain the linkage peak (Additional file 2: Table S7). The highest LOD score obtained for FEV_1_/FVC was 3.29 localized to 219 cM in chromosome 2 (chr 2: 217963480 bp – 218313210 bp) (Figure 2). This locus did not remain significant (LOD =2.2) after adjustment for 10 GWAS SNPs located between 217 cM −236 cM nominally associated with FEV_1_/FVC (p < 0.001) (Additional file 2: Table S7) and was almost completely explained (LOD: 1.01) by adjustment of GWAS SNPs (n = 38) in a broad region from 200–250 cM that were associated with FEV_1_/FVC (p < 0.001) (Additional file 2: Table S7). Further adjustment for additional covariates such as age^2^, height^2^, BMI, for self-reported COPD/asthma and those who took asthma/COPD/bronchitis medications did not significantly change results of the linkage analyses Additional file 1: Figures S3a and S3b.

## Discussion

This study identified a novel linkage peak in chromosome 2 for FEV_1_/FVC and confirmed a previously identified linkage peak in chromosome 6 for FEV_1_. This study also replicated some of the GWAS SNPs identified in previous studies. Though this study identified SNPs in the *CYP2U1* and *PHACTR2* genes that were associated with FEV_1_, these findings could not be replicated in independent datasets.

Two previous reports identified a linkage peak for FEV_1_ at the same locus (chromosome 6 at 28 cM (~280 kb)) as the present study [32,33]. As compared to previous linkage studies we had improved marker density (average marker spacing = 0.5 cM) and improved accuracy by using haplotype-based IBD estimation. The availability of GWAS data also allowed us to evaluate whether GWAS SNPs under the linkage peak explained the linkage peak identified for FEV_1_. While we found a modest attenuation of the FEV_1_ linkage peak after adjustment for common GWAS SNPs under the linkage peak that were nominally associated with FEV_1_ (p < 0.001), they did not completely explain the linkage peak. In contrast, the novel linkage peak identified in chromosome 2 at 219 cM (~372 kb) for FEV_1_/FVC was almost completely explained, by adjustment for the common GWAS SNPs under the linkage peak that were nominally associated with FEV_1_/FVC (p < 0.001). These findings suggest that common variants alone are insufficient to explain some linkage peaks such as the linkage peak in chromosome 6 for FEV_1_. As shown in other diseases, the inability to identify association under linkage peaks could in part be attributable to the fact that only common variants are examined under the linkage peak whereas the linkage signal could be caused by multiple rare variants with higher penetrance [34]. Hence, the contribution of multiple rare variants with high penetrance under the linkage peak towards FEV_1_ needs to be evaluated in future studies. The linkage peak identified in chromosome 6 at 28 cM (~280 kb) for FEV_1_ does not contain any known genes. However, there are several DNase I hypersensitivity sites and putative transcription factor binding sites (9H3K27Ac marks) that have been identified in cells from pulmonary epithelium and blood vessels derived from the pulmonary artery within this linkage peak (EnCode data) suggesting that regulatory elements in this region may play an important role in determining lung function. The linkage peak for FEV_1_/FVC in chromosome 2 at 219 cM (~373 kb) contains the genomic region that codes for the *DIRC3* gene, a non coding RNA that is involved in the pathogenesis of familial renal cancers (EnCode data). Though *DIRC3* is expressed in pulmonary tissue its role in determining lung function has not been evaluated. Furthermore, this region also contains DNase I hypersensitivity sites or putative transcription factor binding sites (9H3K27Ac marks) in cells derived from the pulmonary epithelium or vasculature. Thus the linkage peaks identified in this study on chromosomes 2 and 6 may indicate previously unidentified regulatory pathways that may influence longevity through their effect on lung function.

Though this study identified a few novel GWAS SNPs that were associated with FEV_1_ and FEV_1_/FVC, none of these findings could be replicated in the CHARGE/SpiroMeta consortia. However, only 38% of SNPs associated with FEV_1_ or FEV_1_/FVC in LLFS were available for replication in the CHARGE/SpiroMeta consortia. Thus, the remaining SNPs and insertion/deletions polymorphisms associated with FEV_1_ and FEV_1_/FVC in LLFS but not genotyped/imputed in the CHARGE/SpiroMeta consortia will need to be evaluated in future studies. Since the LLFS study participants were not randomly selected to represent the general population but were specifically selected for their family history of exceptional longevity, it is possible that there may be some unique genotypes associated with lung function that may not be replicated in studies that are more representative of the general population. One previous study showed that elderly male offspring (range: 65–89 years) with long lived parents (age at death of at least on parent >80 years) had FEV_1_ that was 330 ml larger than FEV_1_ for male offspring with short lived parents even after controlling for smoking [13]. The findings of the present study along with previous findings [13] might indicate that long lived families have unique genetic variants that contribute to higher lung function among those with exceptional longevity. This study also replicated 12 of 31 GWAS hits for FEV_1_ and FEV_1_/FVC identified in previous meta-analysis (Additional file 2: Tables S5 and S6). The reasons for not confirming all previously identified variants in this study may include limited power, population specific genetic heterogeneity and differences in environmental exposures such as smoking. Genetic heterogeneity in contribution towards determination of lung function in elderly participants (average age among LLFS participants: 68.8 years) as compared to middle aged adults may also contribute to the differences in genetic association observed in the LLFS study as compared to previous studies. As shown in Additional file 2: Tables S3 and S4, several SNPs had highly significant p for heterogeneity between the LLFS and the CHARGE/SpiroMeta dataset supporting the idea that, at least for some loci, heterogeneity in genetic contribution may account for the lack of replication of certain loci.

## Conclusion

The family-based cohort design of the LLFS with extensive genotype information and detailed lung function measurements makes this study a valuable resource to identify genetic determinants of lung function. In addition to confirming some of the previously identified GWAS SNPs and a previously identified linkage peak in chromosome 6 for FEV_1_, this study also identified a novel linkage peak in chromosome 2 for FEV_1_/FVC. Repeated measurements of lung function in this study population along with targeted resequencing under the observed linkage peaks in future studies may help clarify the role of genetic variants in determining preserved lung function among exceptionally long lived individuals.

## Additional files

Additional file 1:
**Supplementary Figures for lung function-RespR.** Figure S1a; Figure S1b; Figures S2a; Figure S2b; Figure S3a; Figure S3b. Quality control for genotyped SNPs; Q-Q plot showing distribution of observed p values for FEV_1_; Q-Q plot showing distribution of observed p values for FEV_1_/FVC; Manhattan plots of GWAS results for FEV_1_; Manhattan plots of GWAS results for FEV_1_/FVC; Linkage peaks on chromosome 6 associated with FEV_1_; Linkage peaks on chromosome 2 associated with FEV_1_/FVC. Additional file 2:
**Table S1.** FEV_1_ GWAS results with suggestive association (p-value<5E-06) from LLFS, regardless of replication or look-up. **Table S2.** FEV_1_/FVC GWAS results with suggestive association (p-value<5E-06) from LLFS, regardless of replication or look-up. **Table S3.** FEV_1_ GWAS results from LLFS with look-up in CHARGE/SPIROMETA consortia and meta-analysis of LLFS and CHARGE/SPIROMETA. **Table S4.** FEV_1_/FVC GWAS results from LLFS with look-up in CHARGE/SPIROMETA consortia and meta-analysis of LLFS and CHARGE/SPIROMETA. **Table S5.** Examination of published association SNPs for FEV_1_ in LLFS FEV_1_ GWAS. **Table S6.** Examination of published association SNPs for FEV_1_/FVC in LLFS FEV_1_ GWAS. **Table S7.** Three GWAS SNPs located between 26 cM-34 cM within the linkage peak in chromosome 6 for FEV_1_; Nineteen GWAS SNPs located between 10 cM-50 cM within the linkage peak in chromosome 6 for FEV_1_; Ten GWAS SNPs located between 217 cM-236 cM within the linkage peak in chromosome 2 for FEV_1_/FVC; Thirty eight GWAS SNPs located between 200 cM-250 cM within the linkage peak in chromosome 2 for FEV_1_/FVC.
