# Supplementary material for: Genetic variants associated with lung function: the long life family study
Source: Respir Res. 2014 Nov 1;15(1):134. doi: 10.1186/s12931-014-0134-x (PMC4228089; doi:10.1186/s12931-014-0134-x)
Supplement: Additional file 1: — Supplementary Figures for lung function-RespR. Figure S1a; Figure S1b; Figures S2a; Figure S2b; Figure S3a; Figure S3b. Quality control for genotyped SNPs; Q-Q plot showing distribution of observed p values for FEV1; Q-Q plot showing distribution of observed p values for FEV1/FVC; Manhattan plots of GWAS results for FEV1; Manhattan plots of GWAS results for FEV1/FVC; Linkage peaks on chromosome 6 associated with FEV1; Linkage peaks on chromosome 2 associated with FEV1/FVC. [file 12931_2014_134_MOESM1_ESM.docx]

**Quality control for genotyped SNPs**

Genotyped SNPs with a call rate <98% were removed by our quality control procedures (n=83,774 SNPs). We also excluded SNPs from certain chromosomal regions such as 2q21, 2q21.1, HLA1 and HLA (chromosome 6), 8p23.1, 8p23 and 17q21.31 which included highly polymorphic regions and inversions that may influence the PC analysis. Genotyped and imputed SNPs were combined into a hybrid dataset, containing a total of 38,245,546 SNPs, of which 2,225,338 SNPs were genotyped and 36,020,208 SNPs were imputed.

**Supplementary Figure 1a:** Q-Q plot showing distribution of observed p values for FEV_1_ among LLFS participants after excluding SNPs with minor allele frequency < 1%, Hardy-Weinberg p < 1.0E-06 and imputed SNPs with r^2^<0.3.


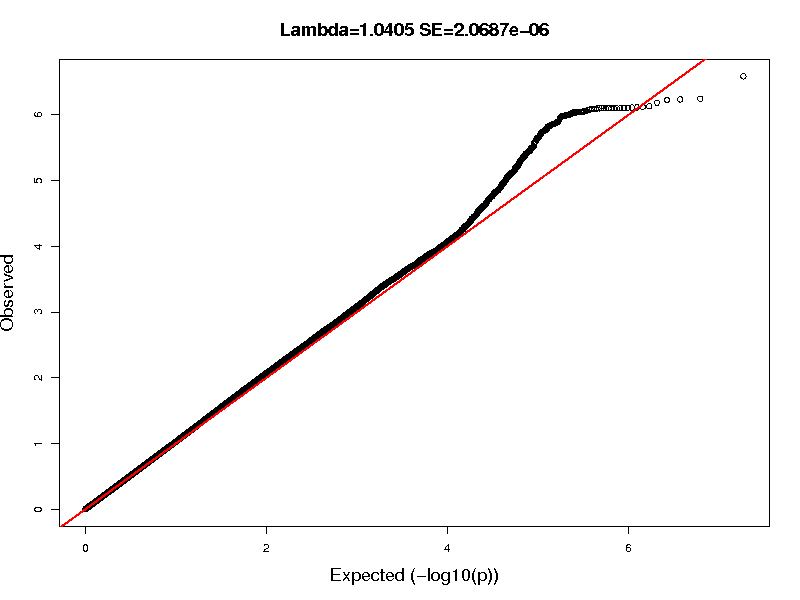


**Supplementary Figure 1b:** Q-Q plot showing distribution of observed p values for FEV_1_/FVC among LLFS participants after excluding SNPs with minor allele frequency < 1%, Hardy-Weinberg p < 1.0E-06 and imputed SNPs with r^2^<0.3.


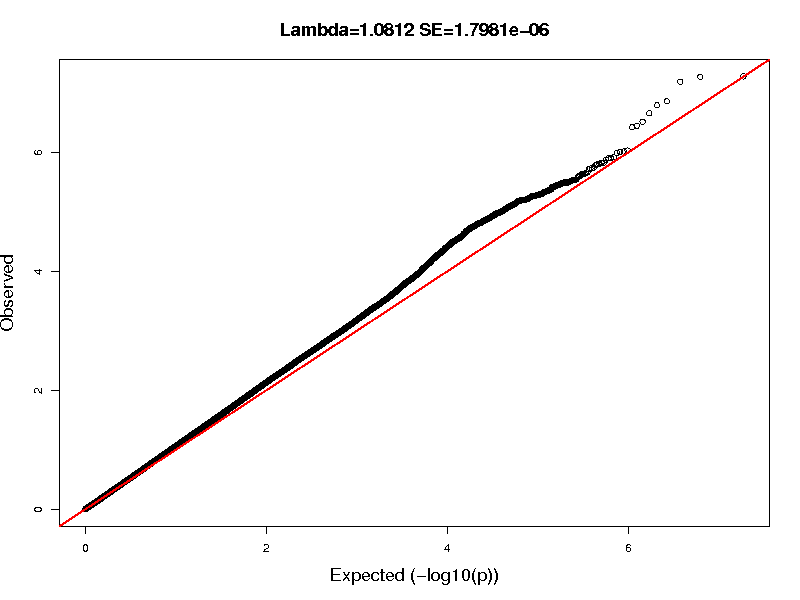


**Supplementary figures 2a:** Manhattan plots of GWAS results for FEV_1_. Horizontal dotted reference lines denote –log_10_(5E-08) and –log_10_(5E-06) for GWA significant and borderline association criteria, respectively. Only SNPs with minor allele frequency > 1% Hardy-Weinberg p > 1.0E-06 and imputed SNPs with r^2^>0.3 were included in these analyses.


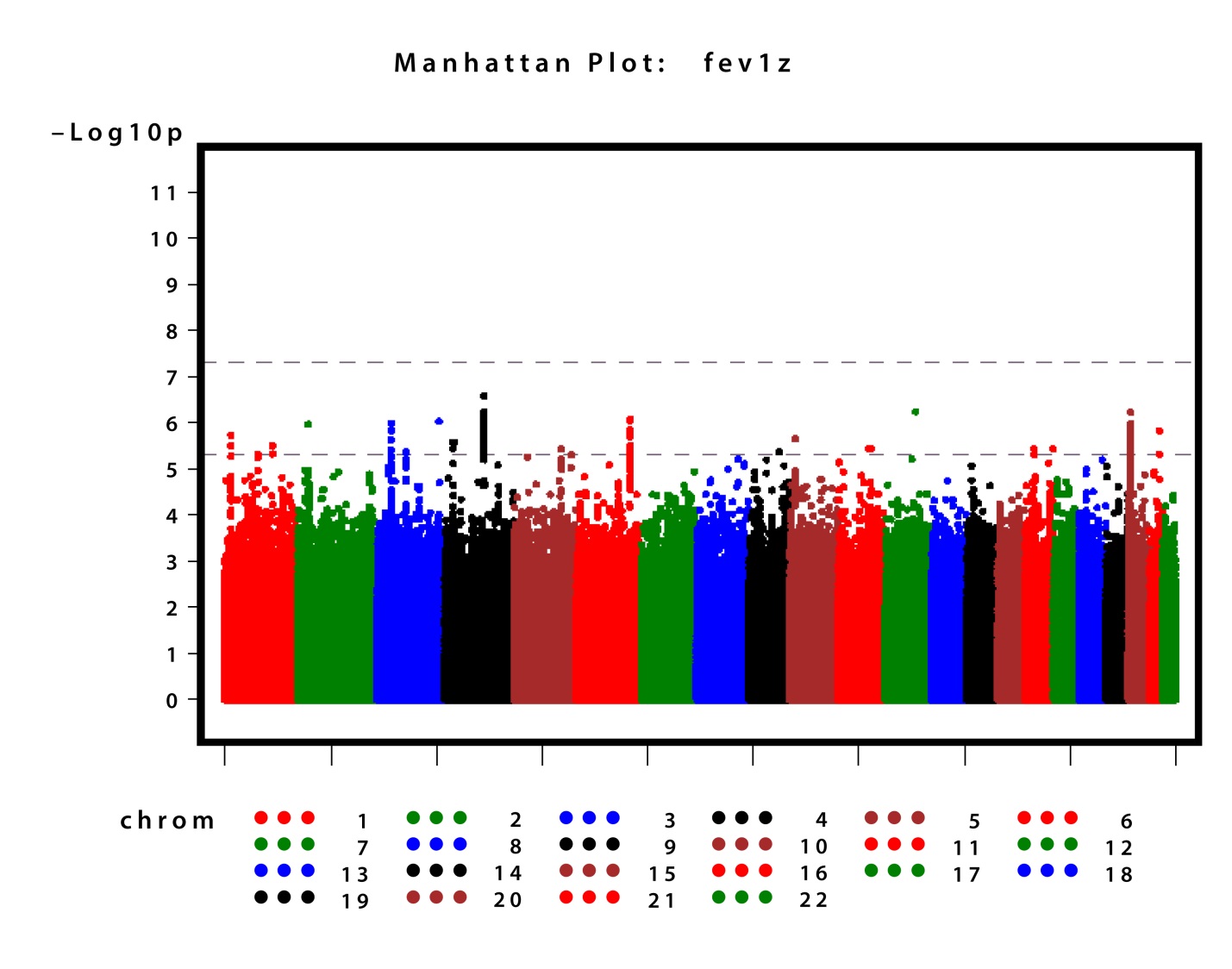


**Supplementary figure 2b:** Manhattan plots of GWAS results for FEV_1_/FVC. Horizontal dotted reference lines denote –log_10_(5E-08) and –log_10_(5E-06) for significant and borderline association criteria, respectively. Only SNPs with minor allele frequency > 1%, Hardy-Weinberg p > 1.0E-06 and imputed SNPs with r^2^>0.3 were included in these analyses.


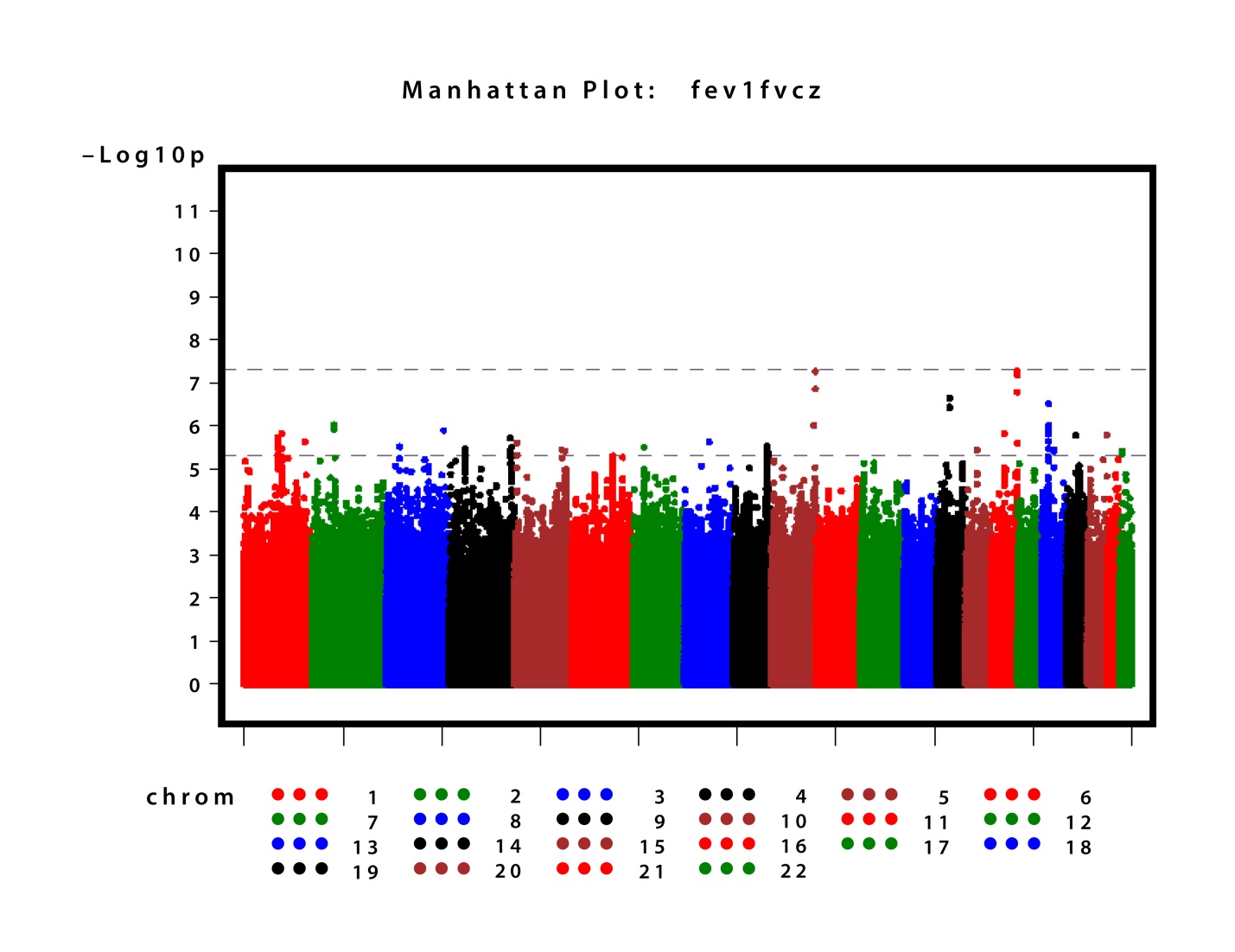


**Supplementary Figure 3a:** Linkage peaks on chromosome 6 associated with FEV_1_ before and after adjustment for GWAS SNPs under the linkage peak associated with FEV_1_ after adjustment for covariates that included age, age^2^, sex, height, height^2^, BMI, study center and ancestry principal components (PC1-20), smoking status (current, never, former), packyears, self reported COPD/asthma and those who take COPD/asthma/bronchitis medications. Original linkage is in red, the linkage in blue is for adjustment of GWAS SNPs (n=7) in a narrow region from 26-34 cM that were associated with p<1E-03, and green is the linkage after adjustment of GWAS SNPs (n=23) in a broad region from 10-50 cM that were associated with p<1E-03.

**Supplementary Figure 3b**: Linkage peaks on chromosome 2 associated with FEV_1_/FVC before and after adjustment for GWAS SNPs under the linkage peak associated with FEV_1_/FVC after adjustment for covariates that included age, age^2^, sex, height, height^2^, BMI, study center and ancestry principal components (PC1-20), smoking status (current, never, former), packyears, self reported COPD/asthma and those who take COPD/asthma/bronchitis medications.. Original linkage is in red, the linkage in blue is for adjustment of GWAS SNPs (n=14) in a narrow region from 217-236 cM that were associated with p<1E-03, and green is the linkage after adjustment of GWAS SNPs (n=44) in a broad region from 200-250 cM that were associated with p<1E-03.

**Funding:**

The Long Life Family Study was supported by the National Institute on Aging (NIA) at the National Institute of Health (U01-AG023712, U01-AG023744, U01AG023746, U01-AG023749, U01-AG023755, U19-AG023122, K24-AG025727, R01-AG032319), the Glenn Medical Research Foundation, and the National Heart Lung Blood Institute (NHLBI) at the National Institute of Health (R21-HL114237). The content is solely the responsibility of the authors, and does not necessarily represent the official views of the NIA or the National Institutes of Health (NIH).

**Acknowledgments:** The investigators thank LLFS participants and staff for their valuable contributions.
